# Preventing postoperative cognitive dysfunction using anesthetic drugs in elderly patients undergoing noncardiac surgery: a systematic review and meta-analysis

**DOI:** 10.1097/JS9.0000000000000001

**Published:** 2023-01-27

**Authors:** Kuan Zeng, Jingyi Long, Yi Li, Jichang Hu

**Affiliations:** a Department of Psychiatry, Wuhan Mental Health Center; b Affiliated Wuhan Mental Health Center, Tongji Medical College of Huazhong University of Science and Technology; c Research Center for Psychological and Health Sciences, China University of Geosciences; d Department of Pathophysiology, School of Basic Medicine, Key Laboratory of Education Ministry of China for Neurological Disorders, Tongji Medical College, Huazhong University of Science and Technology, Wuhan, China

**Keywords:** elderly, network meta-analysis, noncardiac surgery, postoperative cognitive dysfunction, randomized controlled trials

## Abstract

Postoperative cognitive dysfunction (POCD) is a common neurological system disorder in surgical patients. The choice of anesthetic can potentially reduce POCD. The authors performed this network meta-analysis to compare different anesthetic drugs in reducing the incidence of POCD for elderly people undergoing noncardiac surgery. We searched MEDLINE, EMBASE, the Cochrane Library, and the Web of Science for randomized controlled trials comparing the different anesthetic drugs for noncardiac surgery in elderly from inception until July, 2022. The protocol was registered on the PROSPERO database (CRD#42020183014). A total of 34 trials involving 4314 patients undergoing noncardiac surgery in elderly were included. The incidence of POCD for each anesthetic drug was placebo (27.7%), dexmedetomidine (12.9%), ketamine (15.2%), propofol (16.8%), fentanyl (23.9%), midazolam (11.3%), sufentanil (6.3%), sevoflurane (24.0%), and desflurane (28.3%). Pairwise and network meta-analysis showed dexmedetomidine was significantly reducing the incidence of POCD when compared with placebo. Network meta-analysis also suggested dexmedetomidine was significantly reducing the incidence of POCD when compared with sevoflurane. Sufentanil and dexmedetomidine ranked the first and second in reducing the incidence of POCD with the surface under the cumulative ranking curve value of 87.4 and 81.5%. Sufentanil and dexmedetomidine had the greatest possibility to reduce the incidence of POCD for elderly people undergoing noncardiac surgery.

Highlights

- Dexmedetomidine was significantly reducing the incidence of postoperative cognitive dysfunction (POCD).
- Propofol was significantly reducing the incidence of POCD.
- Sufentanil had the greatest possibility to reduce the incidence of POCD.
- Sevoflurane was the worst sedative agents in reducing the incidence of POCD.

## Introduction

Postoperative cognitive dysfunction (POCD) is a common delayed neurocognitive recovery in elderly patients after surgery^1^,^2^. Study reported that the incidence of POCD in elderly patients after noncardiac major surgery was 25.8%^3^. POCD can cause an increase in postoperative complications, prolong patients’ hospital stays, and increase social medical expenditures. In addition, patients with POCD lose the ability to take care of themselves due to changes in personality, social skills, and cognitive abilities and skills, causing a certain economic burden on families and society^4^. Therefore, the onset of POCD should attract sufficient attention from clinicians. The pathogenesis of POCD is currently unknown and may be related to patient age, surgery, and anesthetic drugs. This study suggests that anesthetic drugs may promote the development of POCD in patients^5^. Therefore, the selection of effective and appropriate anesthetic drugs in noncardiac surgery is of great significance to prevent the occurrence of POCD.

The mechanism of action of anesthetic drugs is mainly enhancement of γ-aminobutyric acid (GABAA) receptors activating c1-channels and direct activation of GABAA receptors, enhancement of inhibitory postsynaptic potentials, antagonism of *N*-methyl-d-aspartate effects. And GABAA and *N*-methyl-d-aspartate receptor actions are closely related to the formation of cognitive functions such as learning memory^6^,^7^. GABA levels may drop abruptly with the discontinuation of postoperative sedation, then could result in POCD^8^. Current clinical evidence also suggests that the incidence of POCD varies depending on the sedation agent used. Anesthesia providers have multiple sedation options, and clear evidence-based guidelines are needed to make the best choice for each patient, especially in different clinical situations.

The number of clinical trials and systematic reviews focusing on anesthetic drugs for elderly have been markedly increased, but, to date, no consensus seems to exist. Yu *et al* 9 performed a meta-analysis of 14 studies involving 1626 and indicated that dexmedetomidine was associated with a reduced risk of POCD in elderly. Hovaguimian *et al* 10. performed a meta-analysis of three trials involving 163 patients and indicated that ketamine was associated with a reduced risk of POCD. Chen *et al* 11. performed a meta-analysis of five trials involving 300 patients and indicated that there was no significantly between desflurane and sevoflurane in reducing the incidence of POCD in elderly. Therefore, we performed a network meta-analysis to summarize the current evidence and compare the incidence of POCD for various anesthetic drugs.

## Materials and methods

### Protocol and registration

This work has been reported in line with PRISMA (Preferred Reporting Items for Systematic Reviews and Meta-Analyses), Supplemental Digital Content 1, http://links.lww.com/JS9/A15; Supplemental Digital Content 2, http://links.lww.com/JS9/A16; and AMSTAR (Assessing the methodological quality of systematic reviews), Supplemental Digital Content 3, http://links.lww.com/JS9/A17 Guidelines^12^. The protocol was registered on the PROSPERO database (CRD#42020183014).

### Search strategy

The search strategy aimed to find both published and unpublished studies, including a three-step search strategy that was carried out from inception to July, 2022. An initial limited search of MEDLINE using the keywords: “Postoperative cognitive dysfunction,” “Postoperative decline,” “POCD,” “Postoperative cognitive complication,” “Cognitive function,” “Anaesthetic drugs,” “Anesthetics,” “General Anesthetics,” “Randomized controlled trials,” “RCT,” “elderly,” “Aged,” “older.” Published studies were searched for, including the databases: MEDLINE, EMBASE, the Cochrane Library, and the Web of Science. A full search strategy for the databases is detailed in Appendix I. The following databases were searched to find any unpublished studies: the National Institute of Health Clinical Database. The final step of the search strategy included a review of the reference list of all trials selected for critical appraisal. The search was restricted to papers published in the English language, Supplemental Digital Content 4, http://links.lww.com/JS9/A18.

### Inclusion and exclusion criteria

We searched for randomized controlled trials that investigated the effect of anesthetic drugs in elderly people undergoing noncardiac surgery following the PICOS (Participants, Interventions, Comparisons, Outcomes and Study design) principle. The key search terms included (P) elderly patients (aged ≥60 years); (I) patients were treated by anesthetic drugs; (C/O) the incidence of POCD; (S) randomized controlled trial (RCT). Trials needs to meet the following points: (1) RCT design; (2) articles published in English; (3) studies of elderly (aged ≥60 years) irrespective of ethnicity, gender, and follow-up periods; (4) noncardiac surgery; (5) included the outcomes: the incidence of POCD. Reviews, retrospective studies, observational studies, letters to the editors, and conference abstracts were excluded. Any discrepancies were resolved by discussion with a third author (J.H.).

### Literature screening

Literature retrieval was independently done by the two investigators (K.Z. and J.L.). Disagreement was solved by the third investigator to make a decision (J.H.). Using the PICO framework, our study population (P) of interest was elderly people undergoing noncardiac surgery who had been exposed to any anesthetic drugs (I). Controls (C) comprised individuals with exposure to any anesthetic drugs. We did not exclude those controls who were classified as nonregular users by the authors. The primary study outcome (O) was the incidence of POCD.

### Data extraction

Quantitative data was extracted from all trials included in the review by two independent reviewers (K.Z. and Y.L.). The data extracted included specific details about the type of intervention, populations, context, study design, study methods, and other outcomes of significance to the review question and specific objectives.

### Outcome measures

The outcome was the incidence of POCD.

### Quality assessment

The Cochrane risk of bias tool was used to evaluate the risk of bias for the included studies by the two researchers, independently. Each study was assessed from seven domains. If necessary, the third investigator ought to join the discussions to resolve the disagreement^13^.

### Statistical analysis

Continuous variables were shown by odds ratio (OR) and 95% CI. The heterogeneity was evaluated by the R-4.1.3 software, and the rest was achieved by the Stata12.0 software. Forest plots were generated to determine whether the difference between the pairwise comparisons was statistically significant. The heterogeneity of included RCTs was assessed by the *χ* ^2^ and *I* ^2^ tests. For the *I* ^2^ statistic, the *I* ^2^ value less than 25% was considered low heterogeneity, 25–50% moderate, 50–75% high, and greater than 75% very high heterogeneity. Generally, the *P* value of the *Q* statistic was significant if less than 0.05^14^.

## Results

### Literature search

**Figure 1:**
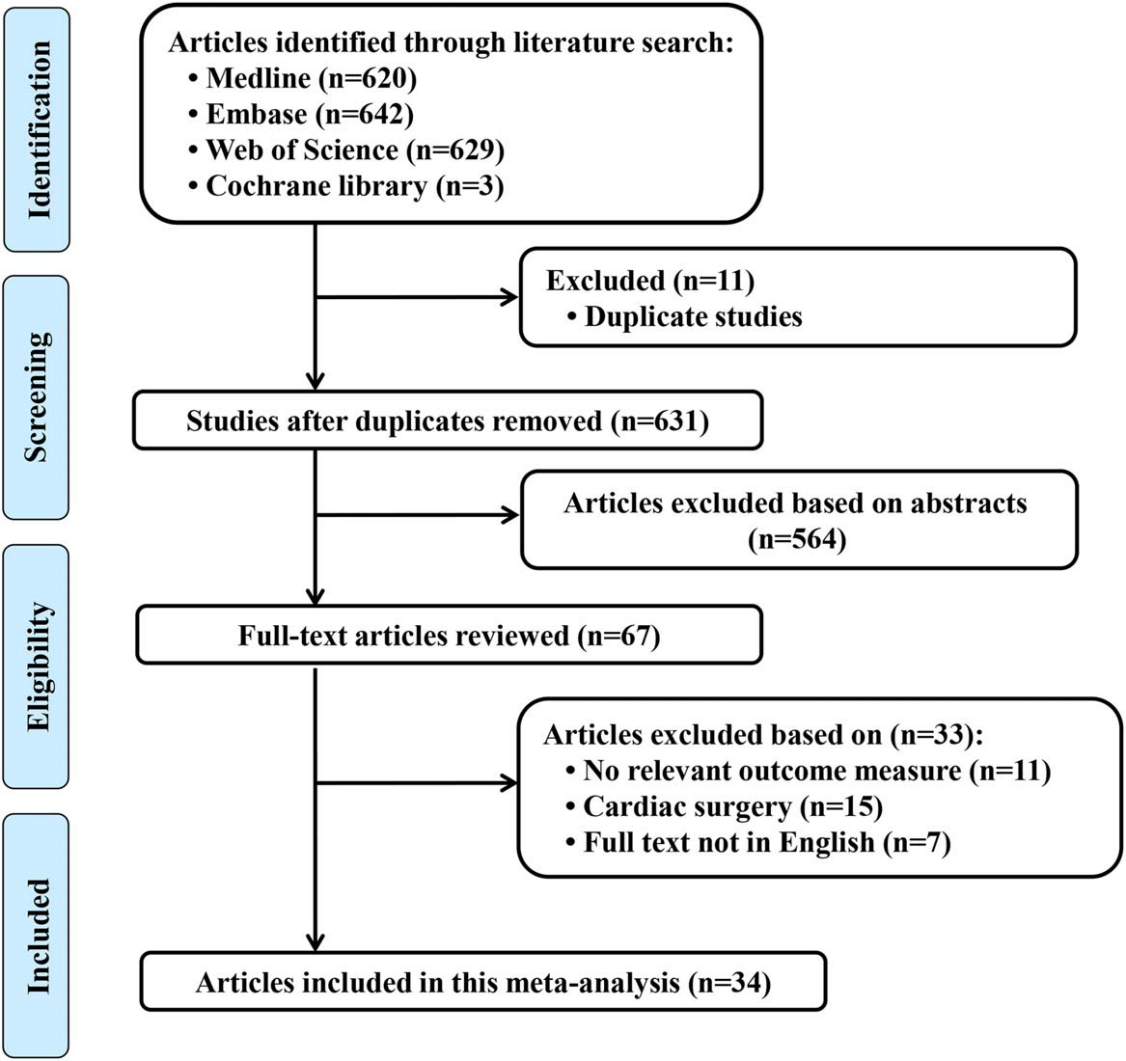
Flow diagram of the study selection process.

We identified 631 articles after excluded duplicate references. Reading the titles and abstracts, excluding 564 references. The remaining 67 references were downloaded and read the full text for further screening, among which 11 references were no relevant outcome measure, 15 were in cardiac surgery, and seven were not in English, and 33 references were excluded, and finally 34 references were included^15^–^48^. Among them, three references compared with three groups, while the others were compared with two groups (Fig. 1).

### Studies characteristics and quality assessment

**Figure 2:**
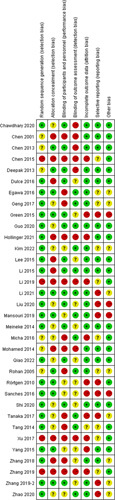
Risk of bias of the included randomized controlled trials (review authors’ judgments about each risk of bias item for each included study. +, low risk; −, high risk; ?, unclear risk.)

**Figure 3:**
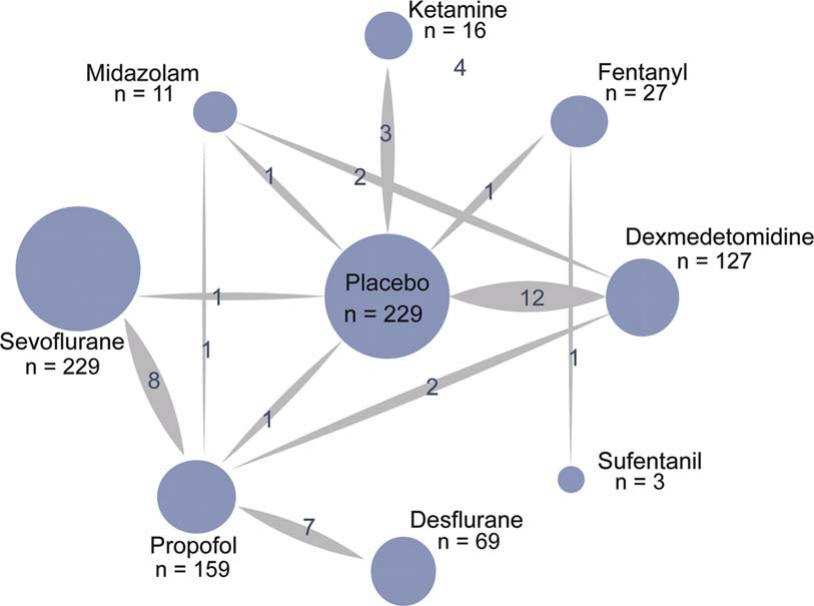
Network of randomized controlled trials comparing different anesthetic drugs in elderly people undergoing noncardiac surgery. The thickness of the connecting lines represents the number of trials between each comparator, and the size of each node corresponds to the number of subjects who received the same pharmacological agent (sample size).

The basic characteristics of the included literatures are shown in Table 1. Interventions include: placebo, dexmedetomidine, ketamine, propofol, fentanyl, midazolam, sufentanil, sevoflurane, and desflurane. We assessed each study from the seven domains, and each domain was judged as low risk of bias, high risk of bias, or unclear risk of bias (Fig. 2). Among them, there were 12 test of dexmedetomidine versus placebo, three test of ketamine versus placebo, one test of propofol versus placebo, one test of fentanyl versus placebo, one test of midazolam versus placebo, one test of sevoflurane versus placebo, two test of dexmedetomidine versus propofol, two test of dexmedetomidine versus midazolam, one test of propofol versus midazolam, one test of fentanyl versus sufentanil, eight test of propofol versus sevoflurane, and seven test of propofol versus desflurane. Placebo was the most connected network node, and most of the network depends on this node, and propofol was the second network node (Fig. 3).

**Table 1:** Characteristics of included studies

		Treatments											
References	Type of surgery	Treatments 1	Age (y)	Female (%)	Cases/*N*	Treatments 2	Age (y)	Female (%)	Cases/*N*	Treatments 3	Age (y)	Female (%)	Cases/*N*
Chawdhary *et al* 17.	Noncardiac surgery	Dexmedetomidine	66.2±6.1	30.0	13/40	Propofol	64.8±3.9	27.5	9/40				
Hollinger *et al* 18.	Noncardiac surgery	Ketamine	73.4±6.1	44.7	10/47	Placebo	74.8±6.6	54.5	6/44				
Rörtgen *et al* 19.	Noncardiac surgery	Desflurane	65.0–75.0	NA	16/40	Sevoflurane	65.0–75.0	NA	19/40				
Rohan *et al* 20.	Cystoscopy	Placebo	67.0–86.0	26.7	1/15	Propofol	65.0–83.0	20.0	7/15	Sevoflurane	67.0–86.0	26.7	7/15
Rascón-Martínez *et al* 32	Ophthalmic surgery	Placebo	>60	50	8/32	Ketamine	>60	57.6	5/33				
Shi *et al* 22.	Lobectomy	Dexmedetomidine	68.7±4.6	0	7/53	Placebo	68.7±3.4	0	19/53				
Xu *et al* 24.	Ovarian cystectomy	Dexmedetomidine	71.9±1.4	100	3/48	Placebo	72.1±2.2	100	10/48				
Zhang *et al* 25	Colorectal cancer	Dexmedetomidine	73.8±14.5	36.2	0/80	Placebo	74.1±13.9	33.3	8/60				
Chen *et al* 26.	Cholecystectomy	Dexmedetomidine	66.2±7.5	44.1	9/59	Placebo	67.9±6.6	50.8	17/63				
Zhang *et al* 25	Open surgery	Fentanyl	70.0±3.1	47.9	11/48	Sufentanil	69.0±2.1	50.0	3/48				
Egawa *et al* 28.	Lung surgery	Propofol	63.0–73.0	31.9	9/60	Sevoflurane	63.0–72.0	45.8	12/58				
Lee *et al* 29.	Orthopedic surgery	Ketamine	68.3±5.3	44.0	1/25	Placebo	68.4±6.5	80.8	8/26				
Guo *et al* 30.	Cancer	Sevoflurane	66.0–74.0	35.0	12/106	Propofol	66.0–72.5	39.3	10/109				
Yang *et al* 31.	H-UPPP surgery	Fentanyl	72.0±4.0	NA	16/65	Placebo	72.0±5.0	NA	17/65				
Valentin	Noncardiac surgery	Dexmedetomidine	60.0–87.0	62.5	13/68	Placebo	60.0–87.0	58.8	36/72				
Mohamed and Shaaban^35^.	Abdominal surgery	Dexmedetomidine	63.9±5.0	20.0	2/25	Placebo	67.8±5.4	0	10/25				
Kim *et al* 36.	Shoulder surgery	Dexmedetomidine	65.0±5.9	42.5	9/40	Placebo	66.3±6.3	52.5	9/38				
Mansouri *et al* 37.	Cataract surgery	Dexmedetomidine	66.5±1.6	50	6/50	Midazolam	63.6±8.3	50	4/50	Placebo	64.0±7.3	62	10/50
Tang *et al* 38.	Rectal resection	Sevoflurane	70.0±4.3	67.7	33/99	Propofol	69.6±4.8	74.3	30/101				
Li *et al* 40.	Knee or hip surgery	Dexmedetomidine	69.3±7.1	63.6	6/43	Propofol	68.2±6.4	56.4	5/47	Midazolam	66.9±6.6	59.3	7/47
Chen *et al* 11.	Noncardiac surgery	Dexmedetomidine	70.6±4.2	35.6	8/87	Placebo	71.4±4.9	37.7	13/61				
Chen *et al* 43.	Knee or hip surgery	Desflurane	75.0±8.0	42.9	0/35	Sevoflurane	73.0±9.0	48.6	1/35				
Zhang *et al* 44.	Cancer surgery	Propofol	72.8±5.5	30.8	28/189	Sevoflurane	72.4±5.6	33.3	44/190				
Liu *et al* 45.	Radical resection	Dexmedetomidine	69.6±4.4	37.5	3/24	Placebo	68.6±3.9	45.8	6/24				
Geng *et al* 46.	Cholecystectomy	Propofol	≥65	60.0	2/50	Sevoflurane	≥65	56	10/50				
Li *et al* 47.	Cholecystectomy	Dexmedetomidine	69.0±5.0	44.0	10/50	Placebo	70.0±6.0	48.0	21/50				
Deepak *et al* 16.	Noncardiac surgery	Sevoflurane	69.1±4.7	60.0	0/30	Desflurane	69.4±4.4	36.7	1/30				
Micha *et al* 15.	Noncardiac surgery	Propofol	60.0–74.0	NA	1/36	Sevoflurane	60.0–74.0	NA	10/37				
Tanaka *et al* 39.	Knee replacement	Desflurane	69.8±1.2	44.4	26/40	Propofol	70.6±1.4	66.7	19/39				
Zhao *et al* 42.	Noncardiac surgery	Dexmedetomidine	70.0±4.5	47.3	40/315	Placebo	69.2±4.1	42.6	30/101				
Qiao *et al* 23.	Laryngeal surgery	Propofol	≥65	6.3	1/32	Desflurane	≥65	0	3/31				
Green *et al* 33.	Urologic surgery	Desflurane	68.2±6.43	100	6/31	Sevoflurane	67.1±6.11	100	3/26				
Meineke *et al* 34.	Noncardiac surgery	Desflurane	72.3±2.5	64.9	17/37	Sevoflurane	71.9±1.8	70.2	32/47				
Li *et al* 48.	Abdominal surgery	Propofol	62.0–68.0	25.2	38/226	Sevoflurane	62.0–69.0	34.4	46/221				

H-UPPP, H-uvulopalatopharyngoplasty.

### Pairwise meta-analysis

The incidence of POCD for each anesthetic drugs was placebo (27.7%), dexmedetomidine (12.9%), ketamine (15.2%), propofol (16.8%), fentanyl (23.9%), midazolam (11.3%), sufentanil (6.3%), sevoflurane (24.0%), and desflurane (28.3%). Pairwise meta-analysis showed dexmedetomidine was significantly reducing the incidence of POCD when compared with placebo (OR=0.34, 95% CI: 0.26–0.44, *P*<0.001). However, propofol (OR=12.35, 95% CI: 1.27–118.36, *P*=0.030) and sevoflurane (OR=12.35, 95% CI: 1.27–118.36, *P*=0.030) were significantly increasing the incidence of POCD when compared with placebo. In addition, propofol was significantly reducing the incidence of POCD when compared with sevoflurane (OR=0.65, 95% CI: 0.50–0.84, *P*=0.001). Fentanyl was significantly increasing the incidence of POCD when compared with sufentanil (OR=4.46, 95% CI: 1.16–17.18, *P*=0.030) (Table 2).

**Table 2:** Summary odds ratios of the incidence of POCD for elderly people undergoing noncardiac surgery and heterogeneity for each direct comparison

Comparison	Study	OR (95% CI)	P-heterogeneity	*I* ^2^ (%)	*τ* ^2^
Dexmedetomidine vs. Placebo	12	*0.34* (*0.26–0.44)*	0.550	0	<0.001
Ketamine vs. Placebo	3	0.77 (0.38*–*1.57)	0.470	0	0.604
Propofol vs. Placebo	1	*12.35* (*1.27–118.36)*	-	-	0.030
Fentanyl vs. Placebo	1	0.92 (0.42*–*2.03)	-	-	0.840
Midazolam vs. Placebo	1	0.35 (0.10*–*1.20)	-	-	0.094
Sevoflurane vs. Placebo	1	*12.35* (*1.27–118.36)*	-	-	0.030
Dexmedetomidine vs. Propofol	2	1.33 (0.60*–*2.95)	0.452	0	0.489
Dexmedetomidine vs. Midazolam	2	0.95 (0.38*–*2.35)	0.301	6.6	0.906
Propofol vs. Midazolam	1	0.68 (0.20*–*2.32)	-	-	0.538
Fentanyl vs. Sufentanil	1	*4.46* (*1.16–17.18)*	-	-	0.030
Propofol vs. Sevoflurane	8	*0.65* (*0.50–0.84)*	0.271	20.0	0.001
Propofol vs. Desflurane	7	1.04 (0.66*–*1.63)	0.161	35.0	0.867

*P*<0.05 is considered as significance with italic fonts.

### Network meta-analysis

**Figure 4:**
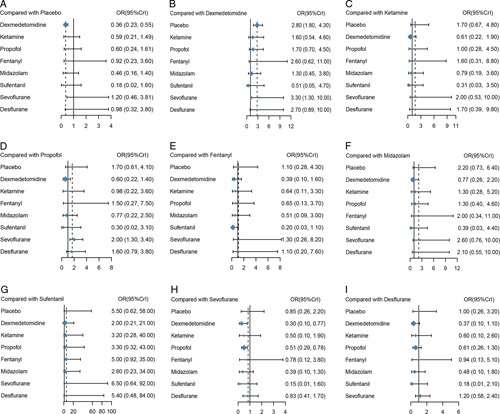
Forest plots of odds ratios (95% creditable intervals) produced by network meta-analysis. OR, odds ratio.

Network meta-analysis indicated dexmedetomidine was significantly reducing the incidence of POCD when compared with placebo (OR=0.36, 95% CI: 0.23–0.55, *P*<0.001). While there was no significant difference in ketamine (OR=0.59, 95% CI: 0.21–1.49, *P*>0.05), propofol (OR=0.60, 95% CI: 0.24–1.61, *P*>0.05), fentanyl (OR=0.92, 95% CI: 0.23–3.60, *P*>0.05), midazolam (OR=0.46, 95% CI: 0.16–1.40, *P*>0.05), sufentanil (OR=0.18, 95% CI: 0.02–1.60, *P*>0.05), sevoflurane (OR=1.20, 95% CI: 0.46–3.81, *P*>0.05), and desflurane (OR=0.98, 95% CI: 0.32–3.80, *P*>0.05) in reducing POCD. Moreover, sevoflurane were significantly increasing the incidence of POCD when compared with dexmedetomidine (OR=3.30, 95% CI: 1.30–10.00, *P*<0.05). In addition, sevoflurane were significantly increasing the incidence of POCD when compared with propofol (OR=2.00, 95% CI: 1.30–3.40, *P*<0.05) (Table 3, Figs. 4A–I).

**Table 3:** Network meta-analysis comparisons

	Placebo	Dexmedetomidine	Ketamine	Propofol	Fentanyl	Midazolam	Sufentanil	Sevoflurane	Desflurane
Placebo	1	*2.80* (*1.80–4.30*)	1.70 (0.67–4.80)	1.70 (0.61–4.10)	1.10 (0.28–4.30)	2.20 (0.73–6.40)	5.50 (0.62–58.00)	0.85 (0.26–2.20)	1.00 (0.26–3.20)
Dexmedetomidine	*0.36* (*0.23–0.55*)	1	0.61 (0.22–1.90)	0.60 (0.22–1.40)	0.39 (0.10–1.60)	0.77 (0.26–2.20)	2.00 (0.21–21.00)	*0.30* (*0.10–0.77*)	0.37 (0.10–1.10)
Ketamine	0.59 (0.21–1.49)	1.60 (0.54–4.60)	1	0.98 (0.22–3.60)	0.64 (0.11–3.30)	1.30 (0.28–5.20)	3.20 (0.28–40.00)	0.50 (0.10–1.90)	0.60 (0.10–2.60)
Propofol	0.60 (0.24–1.61)	1.70 (0.70–4.50)	1.00 (0.28–4.50)	1	0.65 (0.13–3.70)	1.30 (0.40–4.60)	3.30 (0.32–43.00)	*0.51* (*0.29–0.78*)	0.61 (0.26–1.30)
Fentanyl	0.92 (0.23–3.60)	2.60 (0.62–11.00)	1.60 (0.31–8.80)	1.50 (0.27–7.50)	1	2.20 (0.34–11.00)	5.00 (0.92–35.00)	0.78 (0.12–3.80)	0.94 (0.13–5.10)
Midazolam	0.46 (0.16–1.40)	1.30 (0.45–3.80)	0.79 (0.19–3.60)	0.77 (0.22–2.50)	0.51 (0.09–3.00)	1	2.60 (0.23–34.00)	0.39 (0.10–1.30)	0.48 (0.10–1.80)
Sufentanil	0.18 (0.02–1.60)	0.51 (0.05–4.70)	0.31 (0.03–3.50)	0.30 (0.02–3.10)	0.20 (0.03–1.10)	0.39 (0.03–4.40)	1	0.15 (0.01–1.60)	0.18 (0.01–2.10)
Sevoflurane	1.20 (0.46–3.81)	*3.30* (*1.30–10.00*)	2.00 (0.53–10.00)	*2.00* (*1.30–3.40*)	1.30 (0.26–8.20)	2.60 (0.76–10.00)	6.50 (0.64–92.00)	1	1.20 (0.58–2.40)
Desflurane	0.98 (0.32–3.80)	2.70 (0.89–10.00)	1.70 (0.39–9.80)	1.60 (0.79–3.80)	1.10 (0.20–7.60)	2.10 (0.55–10.00)	5.40 (0.48–84.00)	0.83 (0.41–1.70)	1

*P*<0.05 is considered as significance with italic fonts.

### Rank probability

**Figure 5:**
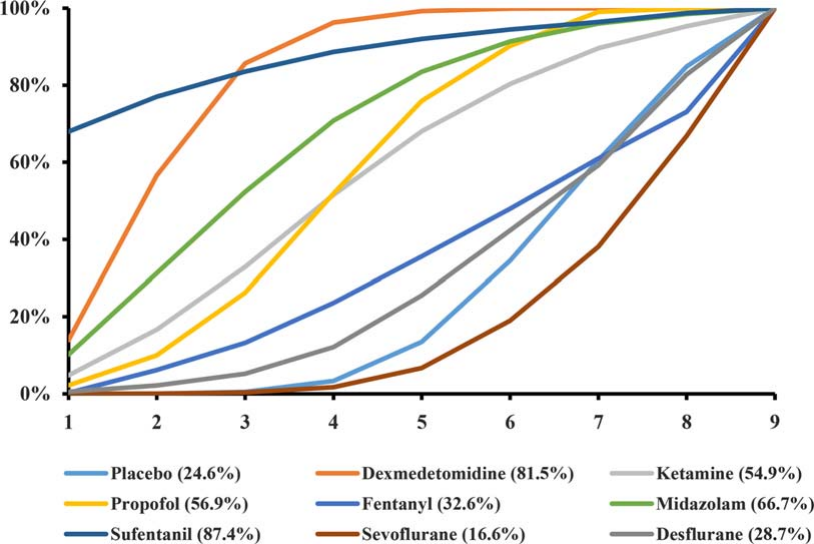
Surface under the cumulative ranking curve, expressed as percentages, ranking the therapeutic effects of reducing the incidence of postoperative cognitive dysfunction for elderly people undergoing noncardiac surgery. For effcacy assessment, the pharmacological agent with the highest surface under the cumulative ranking curve value would be the most efficacious treatment.

The cumulative probabilities of anesthetic drugs in reducing the incidence of POCD for elderly people undergoing noncardiac surgery was shown in Figure 5. The ranking order was as follows: placebo (24.6%), dexmedetomidine (81.5%), ketamine (54.9%), propofol (56.9%), fentanyl (32.6%), midazolam (66.7%), sufentanil (87.4%), sevoflurane (16.6%), and desflurane (28.7%).

### Heterogeneity and inconsistency of included studies

**Figure 6:**
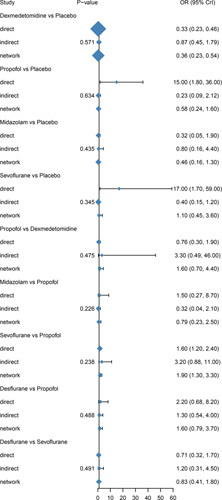
Node splitting results for each comparison. OR, odds ratio.

The node-splitting model was used to assess the inconsistency between direct and indirect evidence. If *P* value was over 0.05, the differences between each direct and indirect comparison were considered nonsignificant. If not, the differences were considered significant. Node-splitting analysis for all the outcomes are shown in Figure 6. As shown in the Figure 6, each *P* value of the node-splitting model was greater than 0.05, indicating that there was no significant difference between the results of direct and indirect comparisons for all the outcomes.

### Comparison-adjusted funnel plots of included studies

**Figure 7:**
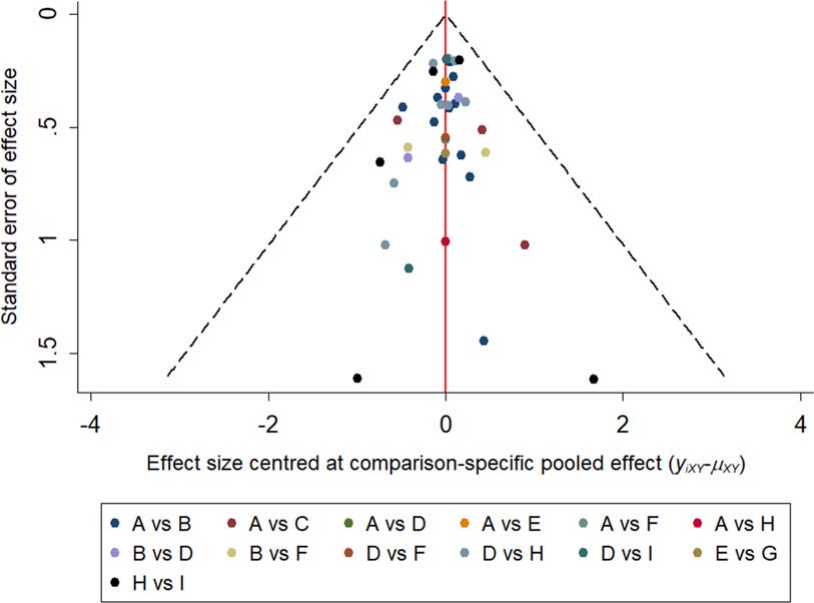
Comparison-adjusted funnel plot for the network meta-analysis. The red line suggests the null hypothesis that the study-specifc effect sizes do not differ from the respective comparison-specifc pooled effect estimates. Different colors represent different comparisons.

The funnel figure was used to evaluate possible publication bias (Fig. 7). All studies were within 95% CI, and the data were both sides of the *X*=0 line, and had good symmetry to the funnel plot, indicating that there had no selectivity and publication bias, and there were two scatter points were located at the bottom of the funnel plot, indicating the presence of small sample effects.

## Discussion

This meta-analysis, which included 34 trials with 4314 undergoing noncardiac surgery in elderly patients. Based on the Bayesian network meta-analysis method, we compared the efficacy of placebo, dexmedetomidine, ketamine, propofol, fentanyl, midazolam, sufentanil, sevoflurane, and desflurane in reducing the incidence of POCD for elderly people undergoing noncardiac surgery. In order to provide a reference for the selection of anesthetic drugs for elderly people undergoing noncardiac surgery. The major results of our study were summarized as follows. First, patients on sufentanil had the lowest incidence of POCD, followed by those on midazolam and dexmedetomidine. Second, sufentanil and dexmedetomidine ranked the first and second in reducing the incidence of POCD, and sevoflurane ranked the last in reducing the incidence of POCD.

Tan *et al* 49. showed that the incidence of POCD in elderly patients was on average 47%. Shoair *et al* 50. showed that even at 3 months after major noncardiac surgery, 15.9% of elderly patients still exhibited POCD. The results of this study showed a 6.3%–28.3% incidence of POCD in elderly people undergoing noncardiac surgery, and the results were similar to the relevant literature. Traumatic stress may be one of the important factors contributing to the occurrence of POCD in elderly surgical patients. Controlling surgical traumatic stress is an important requirement for clinical anesthesia, and exploring safe and effective anesthetic drugs is important to improve the quality of anesthesia and reduce the occurrence of POCD.

Dexmedetomidine is a highly selective α2-adrenoceptor agonist that provides good sedation and analgesia in surgical patients without causing respiratory depression, but is prone to hypotension and bradycardia.Dexmedetomidine inhibits the release of proinflammatory cytokines such as interleukin (IL)-6 and tumor necrosis factor (TNF)-α in a model of cerebral ischemia, producing neuroprotective effects^51^. Dexmedetomidine offers a new option for the clinical prevention and treatment of POCD, with promising applications. Clinical studies have reported the role of dexmedetomidine in the prevention of POCD^52^,^53^, however, other clinical studies have also found that intraoperative use of dexmedetomidine does not prevent the occurrence of POCD^54^. In this study, base on 12 trials, the results showed dexmedetomidine was significantly reducing the incidence of POCD when compared with placebo, while other anesthetic drugs cannot reduce the incidence of POCD when compared with placebo. The reason for this phenomenon is the lack of more comparisons between anesthetic drugs and placebo. An RCT-based NMA may imply the most objective comparison of the same endpoint. We performed an NMA, which allows the creation of multiple treatment comparisons, allowing us to synthesize data with not only direct evidence (within-trial comparisons) but also indirect evidence (between-trial comparisons by co-comparing treatments). In addition, the rank probabilities indicate that sufentanil and dexmedetomidine ranked the first and second in reducing the incidence of POCD in elderly people undergoing noncardiac surgery.

Although the mechanism by which dexmedetomidine reduces POCD has not been determined, there are at least two theories that could explain the potential mechanism. Aβ, TNF-α, IL-1β, and IL-6 not only accurately respond to brain inflammatory response in the brain, but also closely correlates with the occurrence and development of POCD. During the inflammatory phase of the central nervous system, TNF-α, and IL-1β can facilitate the migration of peripheral inflammatory factors, promote excitatory neurotoxic injury, amplify the central nervous system inflammatory response, and participate in the development of POCD^55^. Dexmedetomidine promotes the secretion of anti-inflammatory factor IL-10 and inhibits the production of TNF-α and IL-1β, thus adjusting the balance of systemic proinflammation and anti-inflammation. On the one hand, dexmedetomidine acts on α2 adrenoceptors in the central and peripheral nervous system, inhibiting sympathetic activity, reducing stress response, and activating cholinergic anti-inflammatory pathways, thus reducing the intensity of systemic inflammatory response^51^,^56^. On the other hand, dexmedetomidine reduces the expression of inflammatory factors by inhibiting Toll-like receptor 4/NF-κB, JAK2-STAT3, and NF-κB/COX-2 pathways. COX-2 pathway to downregulate NF-κB pathway activity, thereby reducing the expression of inflammatory factors^57^. Dexmedetomidine also improved lipopolysaccharide-induced neuronal apoptosis by downregulating the expression of B-cell lymphoma/leukemia-2 gene (Bcl-2)-related proteins and upregulating the expression of antiapoptotic proteins, suggesting that the reduction of neuronal autophagy caused by dexmedetomidine may be related to the improvement of cognitive dysfunction^58^.

Sufentanil is a commonly used opioid analgesic in clinical practice. It has been shown that sufentanil can inhibit the release of inflammatory factors and reduce the stress response. In a study on the mechanism of ischemia-reperfusion lung injury in rat limbs by sufentanil pretreatment, it was shown that sufentanil could inhibit the production of inflammatory mediators by inhibiting NF-κB expression in lung tissues and blocking the initiation of ischemia-reperfusion injury in limbs upstream^59^. Similarly, several studies also found that sufentanil significantly inhibited TNF-α and IL-1β expression^60^. However, there is only one study on the effects of sufentanil in elderly people undergoing noncardiac surgery. Therefore, more randomized controlled studies with high quality, large number of samples and strict design should be designed to explore the role of sufentanil in elderly people undergoing noncardiac surgery.

Sevoflurane is a commonly used inhalational anesthetic in clinical practice. Studies have shown that sevoflurane inhalation leads to activation of hippocampal microglia and POCD in aged rats. S100A8 protein is a proinflammatory factor that activates the Toll-like receptor 4 pathway, leading to microglia activation, increased expression of inflammatory factors, and production of POCD^61^. In this study, sevoflurane can the increase incidence of POCD when compared with placebo, and sevoflurane is the worst drug in reducing the incidence of POCD in elderly people undergoing noncardiac surgery.

The advantage of our study depended on the comprehensive evaluation and ranking of the efficacy of placebo, dexmedetomidine, ketamine, propofol, fentanyl, midazolam, sufentanil, sevoflurane, and desflurane reducing the incidence of POCD for elderly people undergoing noncardiac surgery, which had certain guiding significance for clinicians to treat elderly patient with noncardiac surgery. This systematic review included several limitations. First, there were relatively few direct comparisons between different drug trials, this makes it difficult to draw accurate results for direct comparisons. Second, some studies were small-scale, with too few subjects, which may lead to bias, and the time points of all the outcome indicators were not the same. There may be subjective reasons for the evaluation of outcome measure scale, which was easily affected by individual subjective factors. Importantly, POCD development is related with patient-related, surgery-related, and anesthesia-related risk factors. Moreover, a recent study by Glumac *et al* 62. showed that preoperative administration of dexamethasone ameliorates inflammatory response induced by cardiac surgery, and thereby reduced the incidence and severity of POCD following surgery. These also need to be investigated in future studies.

## Conclusion

Taken together, based on the results of this study, we conclude that sufentanil and dexmedetomidine had the greatest possibility to reduce the incidence of POCD in elderly people undergoing noncardiac surgery. While, sevoflurane was the worst anesthetic drugs in reducing the incidence of POCD for elderly people undergoing noncardiac surgery. But because of the short comings of this study, therefore, more randomized controlled studies with high quality, large number of samples, and strict design should be designed to improve the quality of research literature, so as to provide more convincing evidence-based medical evidence and further clarify the efficacy of sufentanil and dexmedetomidine in reducing the incidence of POCD in elderly people undergoing noncardiac surgery.

## Ethical approval

This is a network meta-analysis article with no ethical requirements.

## Sources of funding

This work was supported by grants from National Natural Science Foundation of China (81801077).

## Authors’ contribution

K.Z., Y.L., J.H., and J.L. conceived and designed the study. K.Z., Y.L., and J.L. performed the literature search. K.Z., Y.L., and J.L. were involved in the data collection and interpretation. K.Z., Y.L., and J.L. were involved in the data analysis. K.Z., Y.L., and J.L. were involved in the data interpretation. J.H. drafted the manuscript. K.Z., Y.L., J.H., and J.L. accessed and verified the underlying data reported in the manuscript. All authors were involved in revising the manuscript and approved the final submitted version.

## Conflicts of interest disclosure

The authors declare that they have no financial conflicts of interest with regard to the consent of this report.

## Research registration unique identifying number (UIN)

None.

## Guarantor

Jichang Hu.

## Data statement

This is a meta-analysis article, data availability is not applicable, please contact the corresponding author if some data needed.

## Provenance and peer review

Not commissioned, internally peer-reviewed.

## Supplementary Material
