# Supplementary material for: Preventing postoperative cognitive dysfunction using anesthetic drugs in elderly patients undergoing noncardiac surgery: a systematic review and meta-analysis
Source: Int J Surg. 2023 Jan 27;109(1):21–31. doi: 10.1097/JS9.0000000000000001 (PMC10389238; doi:10.1097/JS9.0000000000000001)
Supplement: Supplementary file 2 [file js9-109-21-s002.docx]

**Identification of studies via databases and registers**

Records removed *before screening*:

Duplicate records removed (n =11 )

Records marked as ineligible by automation tools (n =0 )

Records removed for other reasons (n =0 )

Records identified from*:

Databases (n =642 )

Registers (n =0 )

**Identification**

Records screened

(n =631 )

Records excluded**

(n =564 )

Reports sought for retrieval

(n = 67)

Reports not retrieved

(n =0 )

**Screening**

Reports assessed for eligibility

(n = 67)

Reports excluded:

**No relevant outcome measure**  (n =11 )

**Cardiac surgery**  (n =15 )

**Full text not in English**  (n = 7)

Studies included in review

(n =34 )

Reports of included studies

(n = 34)

**Included**

*Consider, if feasible to do so, reporting the number of records identified from each database or register searched (rather than the total number across all databases/registers).

**If automation tools were used, indicate how many records were excluded by a human and how many were excluded by automation tools.

*From:*  Page MJ, McKenzie JE, Bossuyt PM, Boutron I, Hoffmann TC, Mulrow CD, et al. The PRISMA 2020 statement: an updated guideline for reporting systematic reviews. BMJ 2021;372:n71. doi: 10.1136/bmj.n71

For more information, visit: <http://www.prisma-statement.org/>
