# Supplementary material for: Preventing postoperative cognitive dysfunction using anesthetic drugs in elderly patients undergoing noncardiac surgery: a systematic review and meta-analysis
Source: Int J Surg. 2023 Jan 27;109(1):21–31. doi: 10.1097/JS9.0000000000000001 (PMC10389238; doi:10.1097/JS9.0000000000000001)
Supplement: Supplementary file 4 [file js9-109-21-s004.doc]

**Supplementary List 1 Search Strategy**

**PubMed**

#1 (((Cognitive Dysfunction*[Title/Abstract]) OR Cognitive Impairments *[Title/Abstract]) OR Neurocognitive Disorder *[Title/Abstract]) OR Cognitive Decline *[Title/Abstract] OR Mental Deterioration *[Title/Abstract]

#2 ((Postoperative *[Title/Abstract]) OR Post-surgical *[Title/Abstract])

#3 #1 AND #2

#4 ((Postoperative Decline *[Title/Abstract]) OR " Postoperative Cognitive Complication *"[Title/Abstract]) OR Postoperative Cognitive Dysfunction *[Title/Abstract] OR "POCD*"[Title/Abstract]) OR Cognitive function *[Title/Abstract]

#5 #3 OR #4

#6 ((((((((((Anesthetic Agents *[Title/Abstract]) OR " Anesthetic Drugs *"[Title/Abstract]) OR " Anesthetic *"[Title/Abstract]) OR " Anesthetic Effect *"[Title/Abstract]) OR " Sedative Agents *"[Title/Abstract]) OR " Anesthetic Gases *"[Title/Abstract]) OR " Inhalation Anesthetics *"[Title/Abstract])

#7 #5 AND #6

**EMBASE**

#1 Anesthetic Agents * AND ([article]/lim OR [article in press]/lim OR [conference abstract]/lim OR [conference paper]/lim) AND ([chinese]/lim OR

[english]/lim) AND [embase]/lim

#2 Anesthetic Drugs * AND ([article]/lim OR [article in press]/lim OR [conference abstract]/lim OR [conference paper]/lim) AND ([chinese]/lim OR [english]/lim) AND [embase]/lim

#3 Anesthetic * AND ([article]/lim OR [article in press]/lim OR [conference abstract]/lim OR [conference paper]/lim) AND ([chinese]/lim OR [english]/lim) AND [embase]/lim

#4 Anesthetic Effect * AND ([article]/lim OR [article in press]/lim OR [conference abstract]/lim OR [conference paper]/lim) AND ([chinese]/lim OR [english]/lim) AND [embase]/lim

#5 Sedative Agents * AND ([article]/lim OR [article in press]/lim OR [conference abstract]/lim OR [conference paper]/lim) AND ([chinese]/lim OR [english]/lim) AND [embase]/lim

#6 Anesthetic Gases * AND ([article]/lim OR [article in press]/lim OR [conference abstract]/lim OR [conference paper]/lim) AND ([chinese]/lim OR [english]/lim) AND [embase]/lim

#7 Inhalation Anesthetics * AND ([article]/lim OR [article in press]/lim OR [conference abstract]/lim OR [conference paper]/lim) AND ([chinese]/lim OR [english]/lim) AND [embase]/lim

#12 #1 OR #2 OR #3 OR #4 OR #5 OR #6 OR #7

#13 Cognitive Dysfunction * AND ([article]/lim OR [article in press]/lim OR [conference abstract]/lim OR [conference paper]/lim) AND ([chinese]/lim OR

[english]/lim) AND [embase]/lim

#14 Cognitive Impairments * AND ([article]/lim OR [article in press]/lim OR [conference abstract]/lim OR [conference paper]/lim) AND ([chinese]/lim

OR [english]/lim) AND [embase]/lim

#15 Neurocognitive Disorder * AND ([article]/lim OR [article in press]/lim OR [conference abstract]/lim OR [conference paper]/lim) AND ([chinese]/lim

OR [english]/lim) AND [embase]/lim

#16 Cognitive Decline * AND ([article]/lim OR [article in press]/lim OR [conference abstract]/lim OR [conference paper]/lim) AND ([chinese]/lim

OR [english]/lim) AND [embase]/lim

#17 Mental Deterioration * AND ([article]/lim OR [article in press]/lim OR [conference abstract]/lim OR [conference paper]/lim) AND ([chinese]/lim

OR [english]/lim) AND [embase]/lim

#18 #13 OR #14 OR #15 OR #16 OR #17

#19 Postoperative AND ([article]/lim OR [article in press]/lim OR [conference abstract]/lim OR [conference paper]/lim) AND ([chinese]/lim OR

[english]/lim) AND [embase]/lim

#20 Post-surgical AND ([article]/lim OR [article in press]/lim OR [conference abstract]/lim OR [conference paper]/lim) AND ([chinese]/lim OR

[english]/lim) AND [embase]/lim

#21 #19 OR #20

#22 #17 AND #21

#23 Postoperative Decline AND ([article]/lim OR [article in press]/lim OR [conference abstract]/lim OR [conference paper]/lim) AND

([chinese]/lim OR [english]/lim) AND [embase]/lim

#24 Postoperative Cognitive Complication* AND ([article]/lim OR [article in press]/lim OR [conference abstract]/lim OR [conference paper]/lim)

AND ([chinese]/lim OR [english]/lim) AND [embase]/lim

#25 Postoperative Cognitive Dysfunction AND ([article]/lim OR [article in press]/lim OR [conference abstract]/lim OR [conference paper]/lim) AND ([chinese]/lim

OR [english]/lim) AND [embase]/lim

#26 POCD AND ([article]/lim OR [article in press]/lim OR [conference abstract]/lim OR [conference paper]/lim) AND ([chinese]/lim

OR [english]/lim) AND [embase]/lim

#27 Cognitive function AND ([article]/lim OR [article in press]/lim OR [conference abstract]/lim OR [conference paper]/lim) AND ([chinese]/lim

OR [english]/lim) AND [embase]/lim

#28 #23 OR #24 OR #25 OR #26 OR #27

#29 #22 OR #28

#30 #12 AND #29
